# Mating Behaviour and Copulatory Mechanism in the Scorpionfly *Neopanorpa longiprocessa* (Mecoptera: Panorpidae)

**DOI:** 10.1371/journal.pone.0074781

**Published:** 2013-09-25

**Authors:** Wen Zhong, Baozhen Hua

**Affiliations:** State Key Laboratory of Crop Stress Biology for Arid Areas, Key Laboratory of Plant Protection Resources and Pest Management, Ministry of Education, Entomological Museum, Northwest A&F University, Yangling, Shaanxi, China; CNRS, France

## Abstract

Sexual conflict during copulation may drive morphological and behavioral evolution in insects. Although nuptial feeding behaviour is well studied in *Panorpa*, whether this behaviour is universal in Panorpidae remains unknown. The scorpionfly *Neopanorpa longiprocessa* Hua & Chou, 1997 was investigated for its mating behaviour, functional morphology of the notal organ, and external genitalia using light microscopy and scanning electron microscopy. The results show that the mating behaviour is not associated with nuptial feeding in *N. longiprocessa*. The morphological basis of this non-nuptial copulation is likely related to the developed notal organ of the male. The notal organ serves a function to seize wings of the female during copulation. Only males that succeed in seizing the female with the notal organ are able to establish genital contact and copulate. The male genitalia exhibit distinct species-specific modification. The epandrium (tergum IX) has evolved a pair of ventral bulbs to grasp the subgenital plate of the female. The hypandrium (sternum IX) has a pair of dorsal processes to control the abdominal end of the female. These results indicate that nuptial feeding is not a universal behaviour in Panorpidae. Presumably, these grasping apparatuses compensate the scorpionflies that fail to provide nuptial gifts, as exemplified by *N. longiprocessa*.

## Introduction

Sexual conflict over control of copulation and fertilization may drive the evolution and diversification of genitalia and non-genital contact structures of insects [1]–[4]. In males, these modified structures are used to grasp, seize, or stimulate the female, and improve male copulation success through overcoming female resistance [5], [6]. These specialized traits diverge rapidly over evolutionary time, and are considered one of the most general evolutionary trends in insects [2]–[4], [7].

Many behavioral adaptations, such as nuptial feeding, are also involved in mediating sexual conflict [8]. Nuptial feeding refers to nutrient transfer from the male to the female during courtship and/or copulation [8], [9]. In insects the nutrient could encompass any form of edible gifts, including preys, glandular products, or even male body [9], [10]. There are two main hypotheses for the function of nuptial gifts: as paternal investment or as mating effort [11], [12]. As mating effort, the nuptial gifts provided by males could enhance male control of copulation by mediating female resistance [8].

Panorpidae in Mecoptera are characterized by various nuptial feeding behaviours in their courtship and copulation [13]–[15]. The males of *Panorpa* benefit from nuptial feeding being increasing their paternity, such as prolonging copulation, increasing sperm transfer rate, decreasing female remating rate, and increasing female egg production [16]–[19]. The mate choice of females is mainly dependant on the males’ ability to offer nuptial gifts [20]–[22].

The scorpionflies in the genus *Panorpa* are often treated as model animals for investigating mating systems of insects [23]–[27]. In general, to enhance male control and overcome female resistance in copulation, *Panorpa* males may offer females a dead arthropod or a salivary secretion as nuptial gift; or they may attempt a coercive mating without nuptial gifts [11], [22], [24]. Recent studies mainly focused on the function and evolution of nuptial feeding behaviours in scorpionflies [28]–[30]. The morphological basis of these behaviours, however, has not attracted sufficient attention.

Various nuptial feeding behaviours are closely related to morphological specializations of the salivary glands of the male. Sexual dimorphism often occurs in the salivary glands of the *Panorpa* species whose male offers salivary secretion(s) to the female as a nuptial gift, the salivary glands being much more prominent in the male than in the female [16], [31], [32]. The well-developed salivary glands evidently provide the morphological basis for the males to secrete glandular masses as a nuptial gift. The structures of the male salivary glands, however, differ markedly both at generic and specific levels [33]. In *Panorpa liui* Hua the salivary glands are devoid of sexual dimorphism, only comprising two simple short secretory tubes in both sexes [34]. Accordingly, the male offers exclusively prey rather than salivary secretions as a nuptial gift to the female. On the other hand, the salivary glands of *Neopanorpa* also consist of only two simple elongate secretory tubes [33]. This renders us to explore the relationship of the mating behaviour and the morphology of *Neopanorpa*.

*Neopanorpa* Weele, the second largest genus in Panorpidae, occurs in the Oriental Region, especially abundant in the southern China and Southeast Asia [35]. Species of *Neopanorpa* are peculiar for their undeveloped salivary glands, developed notal organ, and distinctive male genitalia [33], [36]–[39].

The notal organ is a clamp-like posterior process on tergum III in Panorpidae and is used to grasp the anterior edge of the female’s forewing during copulation [14], [40], [41]. This structure is regarded as an adaptation of *Panorpa* to enhance male control of copulation and to overcome female resistance [24]. It may enable males to prolong mating, especially conducive to coercive copulation [13], [14]. In general, the notal organ is much developed in length in *Neopanorpa* than in *Panorpa* [42].

Genitalia play an important role in sexual interactions in insects [4]. Genitalic structures diverge rapidly over evolutionary time than general body features, often differing greatly among closely related taxa whose general structures differ only slightly [2]–[4], [43], [44]. Current knowledge on the genitalic structures of scorpionflies is mainly concentrated on their values in taxonomy and species delimitation. Their functional morphology has largely been ignored to date [40], [45]–[53], although a preliminary investigation was conducted in *P. jilinensis* [54].

Here two questions arise: why the males of *Neopanorpa* have such an elongated notal organ? And what is the evolutionary significance of the notal organ? In this paper we investigated the mating behaviour and mechanism of *Neopanorpa longiprocessa* Hua & Chou, 1997 through observations of mating behaviour under laboratory conditions and of morphology using light microscopy and scanning electron microscopy.

## Materials and Methods

### Ethics Statement

No specific permits were required for the described field studies: a) no specific permissions were required for these locations/activities; b) locations were not privately-owned or protected; and c) the field studies did not involve endangered or protected species.

### Insect Collection

*Neopanorpa longiprocessa* Hua & Chou is one of the northernmost species of the genus, extending as northward as to the Qinling Mountains of central China [37]. Adults were collected at the Huoditang Forest Farm (33°26′N, 108°26′E, elev. 1590–1830 m) in the Qinling Mountains, Ningshan County, Shaanxi Province, China from late May to August in 2010–2012.

### Insect Rearing

The adults collected were transported to the laboratory in the Huoditang Forest Farm. The rearing method followed Thornhill and Sauer [14] and Engqvist and Sauer [55]. Throughout the experiment, adults were held in screen cages (60×60×80 cm) containing twigs and leaves of plants and moist absorbent cotton. Each cage contained 10 males and 10 females. All individuals were marked on forewings to facilitate identification. The scorpionflies were reared at temperatures of 20±5°C during day-time and 15±3°C during night-time. The relative humidity was 70±5% during day-time and 85±5% during night-time. All the adults were reared under natural photoperiod (L:D = 14 h:10 h). Individuals in the cage were provided with dead beetles (*Anomala* sp. and *Potosia* sp.) and cicada (*Cryptotympana* sp.) as food item. Each individual was supplied 1/2 dead insect per day.

### Mating Behaviour

The mating behaviour was observed every 15 min for one pair of mates till the dawn of the next day (4∶45–5∶15 AM), or the termination of the copulation of all pairs.

The recorded items include the initiation and duration of the mate attraction, the beginning and ending times of the seizing behaviour, and the initiation and duration of copulation. The mating strategy of the male was also recorded.

To determine whether laboratory conditions affect the calling and copulatory behaviours, field observations were added as well.

### Light and Scanning Electron Microscopy

For light microscopy, specimens were fixed *in situ* in Carnoy’s solution (100% ethanol: glacial acetic acid = 3∶1, v/v) for 12 h before being preserved in 75% ethanol. The genitalia were dissected under a Nikon SMZ168 stereoscopic zoom microscope. Some specimens were macerated in cold 10% sodium hydroxide solution for 1–2 h, cleaned in an ultrasonic cleaner for 60 s, and observed under a Nikon SMZ1500 stereo zoom microscope. Photographs were taken with a digital camera attached to the microscope and further treated with the software QImaging Retiga-2000R Auto-Montage.

For scanning electron microscopy (SEM), the ethanol-preserved specimens were dried in a critical-point drier after dehydration in a graded ethanol series, coated with gold in a sputter coater, and examined in a Hitachi S-3400N scanning electron microscope (Hitachi, Tokyo, Japan) at 15 kV.

## Results

### Mating Behaviour

The mating behaviour of *N. longiprocessa* may occur anytime in a day except dawn under laboratory conditions. The mating process can be divided into three phases: calling (mate attraction), seizing, and copulating phases. In all the cases of observation, these phases occur without nuptial feeding. Neither dead insect nor salivary secretion was provided as a nuptial gift. No significant difference of mating behaviour was found between the laboratory and field conditions.

During the calling phase, the male attracts the female through odoriferous communication. The male stretches the hypandrium ventrally to expose the everting paired pouch-shaped pheromone glands in the genital bulb to emit sex pheromones that lure the females nearby, as in *Panorpa* [56]–[58]. Once the female responses to the attraction and approaches to the male, he terminates pheromone emission by reverting the pheromone glands. The everting and reverting of the pheromone glands marked the initiation and termination of the mate attraction. The male approaches the receptive female and pounces on her, curves his abdomen upwards while vibrating wings rapidly, and tries to overlap her wings with his wings. When the female is close enough, the male clamps her wings (usually the right side) with his notal organ, and enters into the seizing phase.

The seizing phase starts when the male holds the wings of the female with his notal organ. During the seizing phase, the male keeps clamping the wings of the female. Then he stretches out his abdomen, and seizes the abdominal end (abdominal segments X and XI), especially the cerci of the female with his hypovalves (sometimes also with gonostyli). After that, the male attempts to pair his genitalia with hers. Usually the female struggles to get rid of the male. The seizing phase could be prolonged and even repeated. This phase can last for hours under laboratory conditions. The duration of the seizing phase is 121.17±41.05 min on average for the 70 pairs of successful copulations.

**Figure 1:**
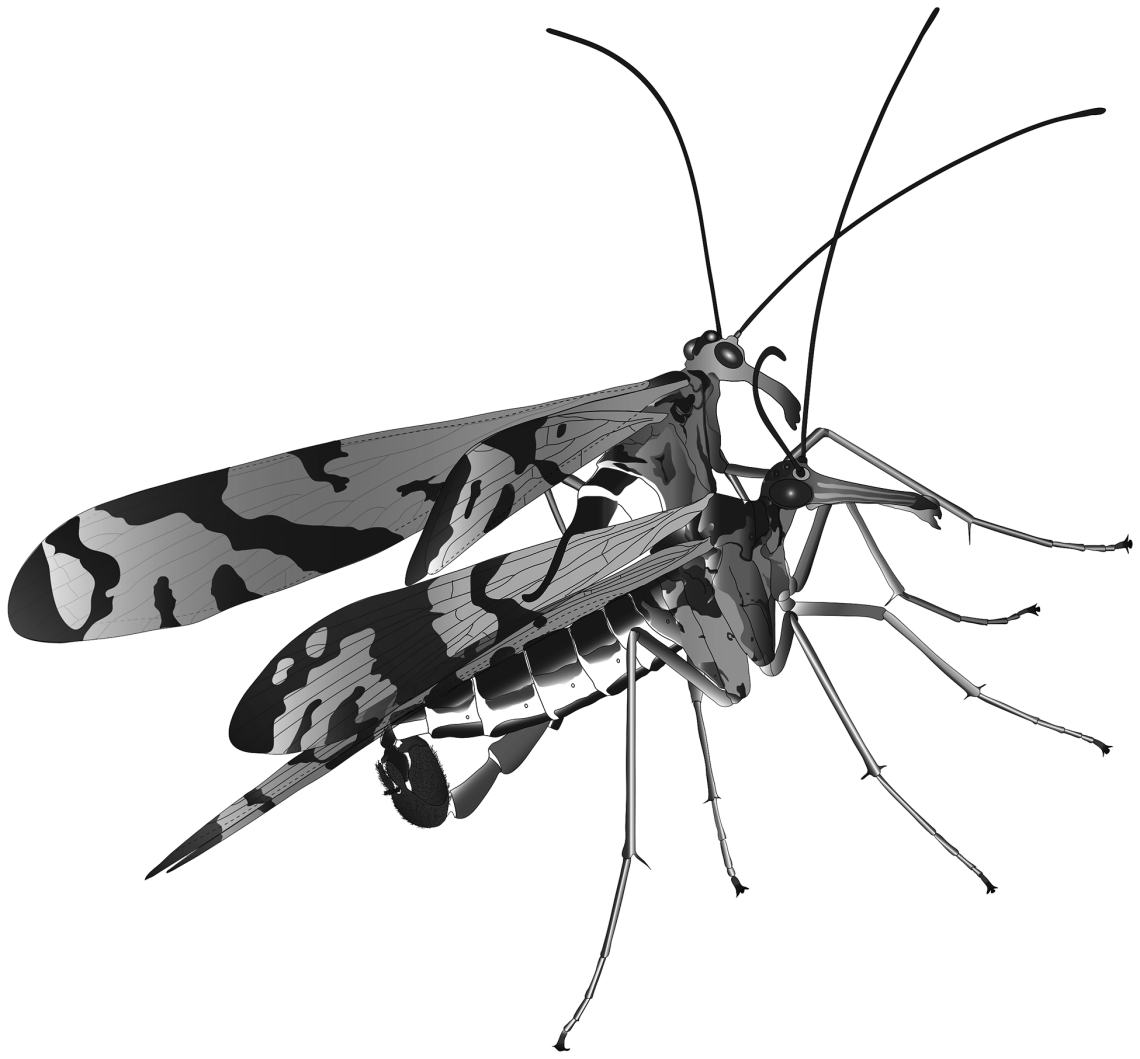
A pair of *Neopanorpa longiprocessa* in copula. The male (the left side) clamps the left wings of the female (the right side) with his notal organ.

**Figure 2:**
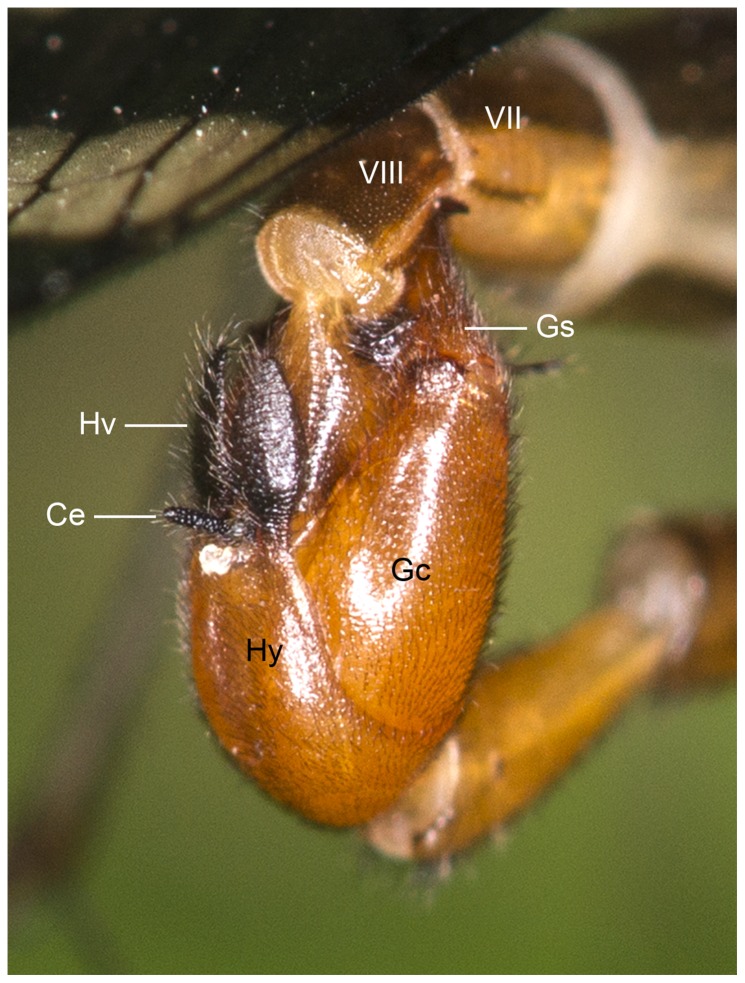
Genitalia of *N. longiprocessa* in copula, with the female at the up and the male at the bottom. Ce, cercus; Gc, gonocoxite; Gs, gonostylus; Hv, hypovalve; Hy, hypandrium.

Once the male has successfully seized the female, he usually attempts to adjust his position to connect their genitalia. The copulating phase starts when the male genital bulb completely encases the female abdominal segments IX–XI (Figures 1 and 2). During copulation, the male keeps clamping the wings of the female from one side with his notal organ (Figure 1) and on the distal abdominal segments with his genital bulb (Figure 2). The coupling pair maintains a V-shaped position (Figure 1).

Sometimes the female twists and/or withdraws her abdomen to try to terminate copulation. Although this resistance can interfere in the copulation, the female is hardly to get rid of the male from steady notal organ clamping. In general, the male will try to reseize the female terminalia with his genitalic clasping structures. The copulation continues after the male reseizes and repositions the female.

The copulating phase terminates when the male loosens his gonostyli and releases his grip on the female abdomen. The copulation duration is 145.64±59.44 min in 70 pairs of successful copulations. Then the male releases the notal organ to free the wings of the female. The female struggles for seconds to disconnect her genitalia from the male’s genital bulb, and then walks away.

### Morphology of the Notal Organ

**Figure 3:**
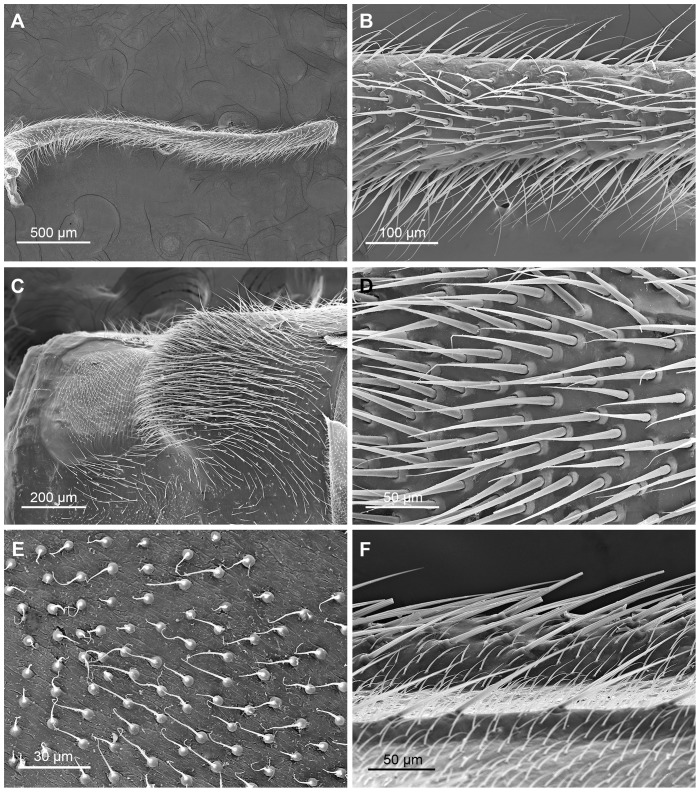
The notal organ and postnotal process of the male and the wing surface of the female in *N. longiprocessa*. (A) Notal organ, lateral view. (B) Magnification of the notal organ in lateral view, showing the setae. (C) Postnotal process, laterodorsal view. (D) Magnification of the postnotal process, showing the setae. (E) Magnification of the dorsal surface of the anterior half of the 4th tergum. (F) Upper surface of the female forewing.

The notal organ of *N. longiprocessa* males is an extremely elongated posterior process on tergum III, extending to the half-way of tergum V, and is used to clamp the wings of the female together with the raised postnotal process on tergum IV during copulation (Figure 1). The ventral surface of the notal organ is densely furnished with pointed long setae, inversed and directed antero-ventrally (Figures 3A and B). The postnotal process of tergum IV is raised dorsad (Figure 3C), and is densely set with thick anterodorsad-directed setae (Figure 3D). The anterior half of tergum IV is weakly sclerotized, and sparsely covered with numerous microtrichia (Figure 3E).

The forewings of the female bear microtrichia on the upper surface and setae along the veins (Figures 3F). These setae interlace with the setae on the notal organ, and impede the female from escaping.

### Genitalic Structures of the Male

Functionally, the male genitalic structures can be divided into interconnecting and grasping structures. The former (penis) is used to connect to the female genitalia during copulation. The latter (including gonostylus, epandrium, and hypandrium) is used to grasp particular part of the female during copulation.

**Figure 4:**
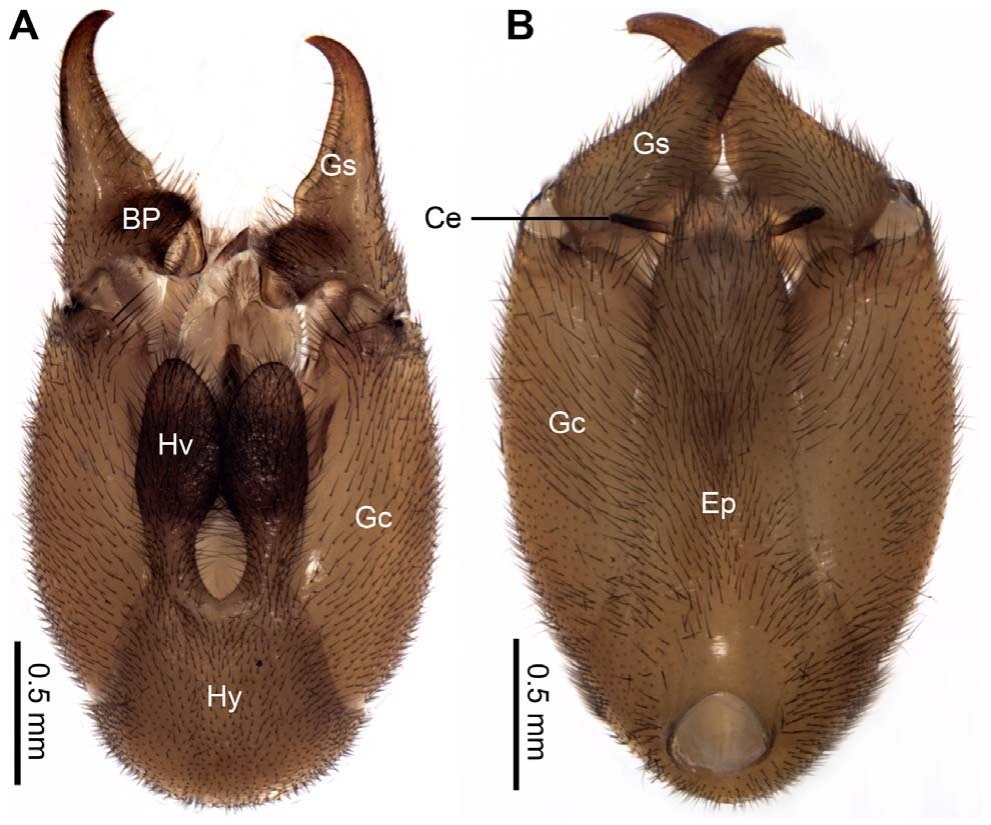
Male genital bulb of *N. longiprocessa*. (A) Ventral view. (B) Dorsal view. BP, basal process; Ce, cercus; Ep, epandrium; Gc, gonocoxite; Gs, gonostylus; Hv, hypovalve; Hy, hypandrium.

**Figure 5:**
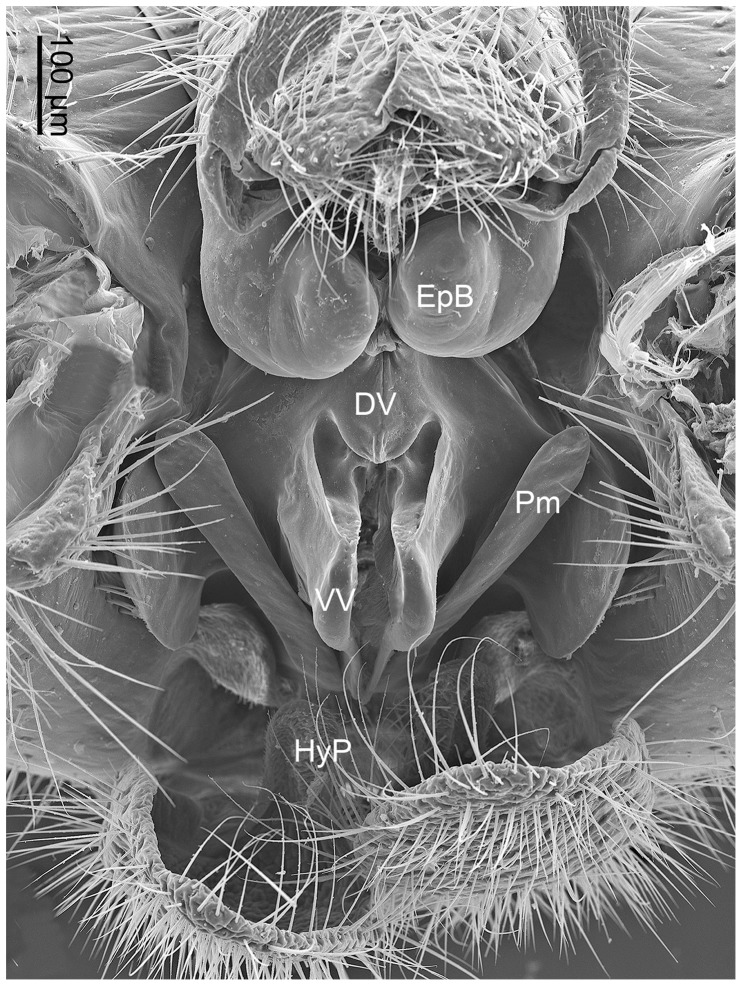
Magnification of the male genital bulb of *N. longiprocessa* in caudal view, with gonostyli removed. DV, dorsal valve of penis; EpB, epandrial bulb; HyP, hypandrial process; Pm, paramere; VV, ventral valve of penis.

The genital bulb of the male *N. longiprocessa* consists of the epandrium (tergum IX), hypandrium (sternum IX), and genitalia. The genitalia consist of paired lateral gonopods and the central valved penis (Figures 4 and 5). The gonopod is two-segmented, comprising the basal gonocoxite and the distal gonostylus (Figure 4).

**Figure 6:**
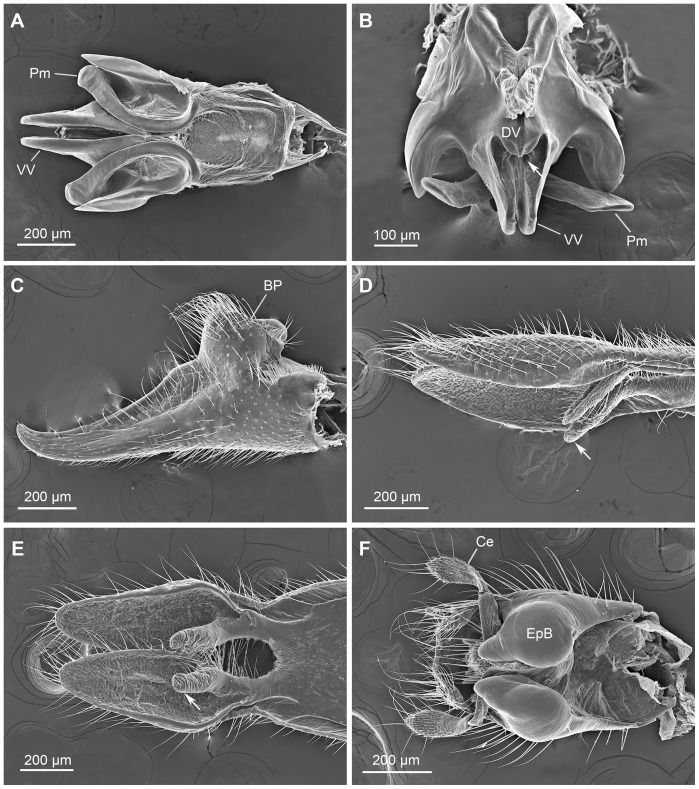
Male genitalic structures of *N. longiprocessa*. (A) Valved penis and parameres, ventral view. (B) Same, caudodorsal view, arrow showing the phallotreme. (C) Left gonostylus, ventral view. (D) Hypovalves, lateral view, arrow showing the hypandrial process. (E) Same, dorsal view, arrow showing the hypandrial process. (F) Apical part of the epandrium in ventral view, showing the epandrial bulb. BP, basal process; Ce, cercus; DV, dorsal valve of penis; EpB, epandrial bulb; Pm, paramere; VV, ventral valve of penis.

The strongly sclerotized penis is composed of bilobed ventral and dorsal valves with the phallotreme situated centrally among the valves (Figures 5, 6A and B). The valved penis is not an intromittent organ and lacks endophallus anatomically (Figures 5, 6A and B).

The paired parameres are basally fused with the penis, consisting of a single basal stalk and paired distal branches (Figure 6A). The ventral branch can stretch ventrally for a limited angle (Figure 6B).

The elongated gonostyli are claspers, grasping the female terminalia during copulation (Figures 1 and 2). Usually, the gonostyli steadily clamp the female’s segment IX at the base, press her body wall to open the genital chamber and to expose the genital plate, at the apex of which is situated the orifice of the spermathecal duct. Each gonostylus has a prominent basal process, which bears long setae, with the median part concaved (Figure 6C). These processes help deform the integument of the female’s genital chamber, impeding the genital plate from retreating (Figure 2).

The hypandrium consists of a broad basal stalk and a pair of slender distal hypovalves. The hypovalves are convex ventrally, with their dorsal surface rough and bearing irregular microsculpturing (Figure 6E). Each hypovalve subbasally has a blunt dorsad-directing process, which bears numerous thick setae on the mesal surface (Figures 6D and E).

The epandrium bears subapically a pair of greatly expanded ventral bulbs, which are weakly sclerotized and produced posteriorly (Figures 5 and 6F). The paired bulbs fill the space between the epandrium and penis (Figure 5).

### Genitalic Structures of the Female

**Figure 7:**
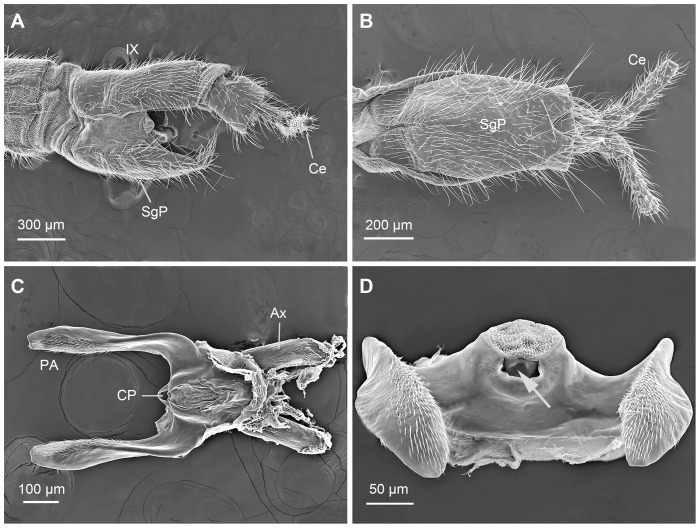
Female genitalia of *N. longiprocessa*. (A) Abdominal segments VIII–XI, lateral view. (B) Abdominal segments IX–XI, ventral view. (C) Genital plate, ventral view. (D) Same, caudal view, arrow showing the copulatory pore (orifice of the spermathecal duct). Ax, axis; Ce, cercus; CP, copulatory pore; PA, posterior arm; SP, subgenital plate.

The genitalia of the female are located on the abdominal segment IX, consisting of a subgenital plate and a genital plate (Figure 7A). The subgenital plate is emarginate distally in a V shape, implying its paired origin (Figure 7B), and curves dorsally from each side to form a shallow genital chamber, in which the genital plate is situated (Figure 7A).

The genital plate is a heavily sclerotized structure, consisting of the main plate and the axis. The main plate extends backward, forming paired posterior arms (Figure 7C). In the axis, the spermathecal duct is fixed in the midventral groove and the irregular-shaped copulatory pore is situated medially at the posterior end (Figure 7D). The genital plate can stretch out to connect her copulatory pore to the phallotreme of the male penis.

## Discussion

To our knowledge, this research is the first attempt to explore the copulatory mechanism in *Neopanorpa* scorpionflies. *N. longiprocessa* is peculiar for the male to seize the female for a long period and not to provide edible nuptial gifts during mating. The developed notal organ of the male plays an important role in the seizing phase. Some specialized structures in the male genitalia, such as epandrial bulbs and hypandrial processes, may be used to counter against the female resistance.

### Co-Evolution of the Mating Behaviour and Male Apparatuses

Male *Panorpa* scorpionflies may obtain mating in three alternative tactics: offering salivary masses, offering dead arthropods, or coercive copulation without nuptial gifts [15], [24]. In *Panorpa* the females mate selectively with males that provide edible gifts, and discriminate against the males that fail to offer nutriments either by rejection or by copulation only for a short time [16], [20]–[22]. Hence, the coercive copulation is considered as the most ineffective tactic because of the female resistance, which causes the shortening of copulation duration and the decreasing of the sperm transfer rate [16], [59].

Coercive copulation is more unusual than the other two tactics in *Panorpa* [55], [60]. In some North American and Japanese species of *Panorpa*, the males usually provide dead arthropods as nuptial gifts. However, the tactic of coercive copulation happens occasionally for the weak or less competitive males [13], [15], [61].

The peculiarity of the mating behaviour in *N. longiprocessa* lies on that the copulation has nothing to do with nuptial feeding. The males of *N. longiprocessa* use the developed notal organ to clamp the wings of the female. Only the male that succeeds in seizing the female can establish genital contact and copulate with her. The seizing phase in *N. longiprocessa* takes almost equal time to copulation. Since the male copulates coercively with the female, he must conquer the resistance from the female. In this case, the male genitalia could contact with the counterparts of the female and keep stability for sperm transfer. This mode of copulation may promote the morphological evolution of the male, and is likely one of the possible reasons for the copulatory apparatuses of *N. longiprocessa* to diverge rapidly from those of *Panorpa* scorpionflies.

Quite different from *Panorpa* scorpionflies with undeveloped notal organs in males [40], [42], the developed notal organ can explain the coercive mating behaviour in *N. longiprocessa*. The notal organ of *N. longiprocessa* is extremely developed, almost corresponding to the width of female’s wings and bear dense setae, both of which likely impede the female wings from withdrawing.

The notal organ of male *Panorpa* scorpionflies is regarded as a modified appendage that can be used in coercive mating behaviours [13], [15]. The function of notal organ was once considered to prolong copulation against female interests [14]. Recent studies, however, show that in some species the organ may not contribute to prolonging copulation [55], [62]. On the other hand, the notal organ exhibits interspecific diversity in the genus *Panorpa* (*cf*. 34,40–42,54), which is likely one of the possible reasons for the difference in ability to prolong copulation.

On the basis of our unpublished comparative study, the developmental degree of the notal organ is negatively correlated with that of the male salivary glands, and vice versa. This phenomenon probably affects the evolution of mating strategy of the males. Males of *P. liui* are characterized by a developed notal organ and a simple undeveloped salivary gland [33], [34], [63], and are unable to generate salivary secretions. Thus the males provide only dead arthropods (prey) as nuptial gifts [34]. Males of the North American *P. nuptialis* and the Japanese *P. japonica* share morphological similarity of notal organ with *P. liui*. Likewise, they produce no salivary secretion for the female to feed upon [40], [61], [64]. The males of *Neopanorpa* usually bear a well-developed notal organ and undeveloped salivary gland [33], [65]. Our result may partly explain the structural reason why the males of *N*. *longiprocessa* do not offer salivary secretion as their nuptial gift: males are too weak with respect to their salivary glands to produce enough saliva offered to females during copulation.

Conflict over control of copulation also drives the evolution of male genitalic structures [7], [44], [66]. The specialized genitalia enhance male ability to control over copulation [3], [4]. In scorpionflies males have evolved specialized genitalia to take advantages in sexual conflict. Their functional morphology has been preliminarily explored in *P. jilinensis* [54]. However, the roles of genitalia in copulation have not been satisfactorily investigated in *Neopanorpa*.

The specialized gonostylus, epandrium, and hypandrium of the male of *N. longiprocessa* are used to grasp particular part of the female abdomen. The gonopods are called as genital claspers [67], indicating their function as claspers to clamp the female during mating. The movable gonostyli, to be more precise, are particularly used as “claspers”. Long setae on the basal process are likely used to increase friction during the seizing phase.

The structure of the male hypovalves in *Neopanorpa* is characterized by the paired hypandrial processes [36], [38]. The modification of this structure leads to the changes of the functions of hypovalves in the courtship and copulation of *N. longiprocessa*. In the seizing period, the male of *N. longiprocessa* uses his hypovalves to entangle the female stretched terminalia and infibulate her cerci. It allows the male to restrict the movement of the female abdomen and easily to approach the female genitalia at the following interactions. During copulation the hypovalves are used to entrap the female’s terminalia not to move till the male releases the female. We speculate that the irregular microsculpturing on the dorsal surface of the hypovalves is likely used to increase friction between the hypovalves and the counterpart of the female.

The epandrial bulb is another specialized genital structure in *N*. *longiprocessa*. According to Miyaké [45], who illustrated the copulatory posture of external genitalia of both sexes in *Panorpa klugi*, we speculate that the epandrial bulb in *N. longiprocessa* likely serves to entrap female’s subgenital plate between the ventral surface of the epandrium and the dorsal surface of the gonocoxites by a specialized genital structure. The moveable apical part of the epandrium could firmly fasten the female terminalia, allowing the male penis to connect with the orifice of the spermathecal duct in the female genital chamber during copulation.

Morphological modification in genitalia and other contact structures is a widespread strategy in male insects to adapt to overcome the female resistance over copulation in sexual conflict [4], [7]. Male strategies aimed at succeeding in copulation can increase mating costs of the female through sexual conflict [9], [68]. The costs, including energetic cost, injury, disease, and predation risk, may cause decrease of female fitness [8], [69]. Thus, the females primarily attempt to decrease or avoid male-imposed mating costs rather than acquire benefits from males [8], [70]. In *N. longiprocessa*, to conquer the female resistance, the male has evolved an extremely elongated notal organ and specialized genitalia, which are used to seize the female during mating. The seized female usually struggles to escape, likely increasing the mating costs through energetic consumption. Due to the increasing mating costs during struggle, the female is likely to yield and accept the copulation. Thus, the non-nuptial feeding mating of *N. longiprocessa* is likely evolved through sexual conflict in scorpionflies. These structural adaptations in *N. longiprocessa* may represent another evolutionary pathway different from *Panorpa* to overcome female resistance.

### Copulatory Mechanism

In general, the male of pterygote insects insert his aedeagus (endophallus) into the reproductive tract of the female during insemination [6]. The male *N. longiprocessa*, however, possesses a valved penis and lacks a median intromittent organ. The male connects his phallotreme directly to the female copulatory pore for sperm transfer, as in *P. jilinensis* [54]. Thus the female is easy to disconnect with the male during copulation. Accordingly, it is the male’s interest to evolve specialized structures to overcome the female resistance and complete the copulation in *Neopanorpa*.

The copulatory mechanism in Panorpidae has only been studied in some species, such as *P. jilinensis* [54], although Miyaké illustrated the function of particular genital structures in *Panorpa klugi* [45]. On the basis of the behavioral observations and the ultrastructure of the external genitalia, we suggest that the copulatory mechanism of *N. longiprocessa* is as follows. Having seized the female with his notal organ, the male adjusts his genitalic position to allow the phallotreme to connect to the copulatory pore (the orifice of the spermathecal duct) of the female for insemination. In order to contact their genitalia, the male uses his gonostyli to seize the female’s terminalia at beginning, then uses its male epandrium to clasp the female subgenital plate, and finally uses its hypovalves to clip the female cerci. These genital structures of the male restrict the movements of the female terminalia, allowing the male to hold the female genitalia tightly against the resistance (Figure 2).

Elucidating copulatory mechanism may help understand the sexual conflict in scorpionflies. In this study, however, detailed genitalic contacts during copulation have not been worked out between the male and female genitalia. The precise copulatory mechanism awaits additional studies using freeze-fixation of copulating pairs, as exemplified by Eberhard & Ramirez [71].

## References

[1] Arnqvist G (1997) The evolution of animal genitalia: distinguishing between hypotheses by single species studies. Biol J Linn Soc 60: 365–379.

[2] Arnqvist G (1998) Comparative evidence for the evolution of genitalia by sexual selection. Nature 393: 784–786.

[3] Eberhard WG (2004) Rapid divergent evolution of sexual morphology: comparative tests of antagonistic coevolution and traditional female choice. Evolution 58: 1947–1970. 15521454 10.1554/04-143

[4] Eberhard WG (2010) Evolution of genitalia: theories, evidence, and new directions. Genetica 138: 5–18. 19308664 10.1007/s10709-009-9358-y

[5] Chapman RF (1998) The insects: structure and function, 4th ed. Cambridge: Cambridge University Press. 770 p.

[6] Gullan PJ, Cranston PS (2010) The insects: an outline of entomology, 4th ed. Oxford: Wiley-Blackwell. 584 p.

[7] Eberhard WG (2004) Male-female conflict and genitalia: failure to confirm predictions in insects and spiders. Biol Rev 79: 121–186. 15005176 10.1017/s1464793103006237

[8] Gwynne DT (2008) Sexual conflict over nuptial gifts in insects. Annu Rev Entomol 53: 83–101. 17680720 10.1146/annurev.ento.53.103106.093423

[9] Vahed K (1998) The function of nuptial feeding in insects: review of empirical studies. Biol Rev 73: 43–78.

[10] Lewis S, South A, Burns R, Al-Wathiqui N (2011) Nuptial gifts. Curr Biol 21: R644–R645. 21920290 10.1016/j.cub.2011.05.046

[11] Engqvist L, Sauer KP (2001) Strategic male mating effort and cryptic male choice in a scorpionfly. Proc R Soc Lond B 268: 729–735. 10.1098/rspb.2000.1423 PMC1088663 11321062

[12] Thornhill R (1976) Sexual selection and paternal investment in insects. Am Nat 110: 153–163.

[13] Thornhill R (1980) Rape in *Panorpa* scorpionflies and a general rape hypothesis. Anim Behav 28: 52–59.

[14] Thornhill R, Sauer KP (1991) The notal organ of the scorpionfly (*Panorpa vulgaris*): an adaptation to coerce mating duration. Behav Ecol 2: 156–164.

[15] Thornhill R (1981) *Panorpa* (Mecoptera: Panorpidae) scorpionflies: systems for understanding resource-defense polygyny and alternative male reproductive efforts. Annu Rev Ecol Syst 12: 355–386.

[16] Sauer KP, Lubjuhn T, Sindern J, Kullmann H, Kurtz J, et al (1998) Mating system and sexual selection in the scorpionfly *Panorpa vulgaris* (Mecoptera: Panorpidae). Naturwissenschaften 85: 219–228.

[17] Engqvist L (2007) Nuptial food gifts influence female egg production in the scorpionfly *Panorpa cognata* . Ecol Entomol 32: 327–332.

[18] Engqvist L (2007) Nuptial gift consumption influences female remating in a scorpionfly: male or female control of mating rate? Evol Ecol 21: 49–61.

[19] Engqvist L, Dekomien G, Lippmann T, Epplen JT, Sauer KP (2007) Sperm transfer and paternity in the scorpionfly *Panorpa cognata*: large variance in traits favoured by post-copulatory episodes of sexual selection. Evol Ecol 21: 801–816.

[20] Kock D, Hardt C, Epplen JT, Sauer KP (2006) Patterns of sperm use in the scorpionfly *Panorpa germanica* L. (Mecoptera: Panorpidae). Behav Ecol Sociobiol 60: 528–535.

[21] Kock D, Sauer KP (2008) Female mating frequency in a wild population of scorpionflies (*Panorpa germanica*, Panorpidae, Mecoptera). J Zool Syst Evol Res 46: 137–142.

[22] Engqvist L (2007) Sex, food and conflicts: nutrition dependent nuptial feeding and pre-mating struggles in scorpionflies. Behav Ecol Sociobiol 61: 703–710.

[23] Carpenter FM (1931) The biology of the Mecoptera. Psyche 38: 41–55.

[24] Byers GW, Thornhill R (1983) Biology of the Mecoptera. Annu Rev Entomol 28: 203–228.

[25] Palmer CM (2010) Diversity of feeding strategies in adult Mecoptera. Terrest Arthrop Rev 3: 111–128.

[26] Engels S, Sauer K (2006) Resource-dependent nuptial feeding in *Panorpa vulgaris*: an honest signal for male quality. Behav Ecol 17: 628–632.

[27] Engqvist L (2007) Male scorpionflies assess the amount of rival sperm transferred by females’ previous mates. Evolution 61: 1489–1494. 17542855 10.1111/j.1558-5646.2007.00107.x

[28] Engqvist L (2011) Male attractiveness is negatively genetically associated with investment in copulations. Behav Ecol 22: 345–349.

[29] Missoweit M, Sauer KP (2007) Not all *Panorpa* (Mecoptera: Panorpidae) scorpionfly mating systems are characterized by resource defence polygyny. Anim Behav 74: 1207–1213.

[30] Missoweit M, Engqvist L, Lubjuhn T, Sauer KP (2008) Nuptial feeding in the scorpionfly *Panorpa vulgaris*: maintenance of genetic variance in sexual advertisement through dependence on condition influencing traits. Evol Ecol 22: 689–699.

[31] Engels S, Sauer KP (2008) A secondary sex trait under construction: age- and nutrition-related salivary gland development in a scorpionfly (Insecta: Mecoptera). J Zool Syst Evol Res 46: 133–136.

[32] Potter E (1938) The internal anatomy of the order Mecoptera. Trans R Entomol Soc Lond 87: 467–501.

[33] Ma N, Liu SY, Hua BZ (2011) Morphological diversity of male salivary glands in Panorpidae (Mecoptera). Eur J Entomol 108: 493–499.

[34] Ma N, Hua BZ (2011) Structural evidence why males of *Panorpa liui* offer prey rather than salivary mass as their nuptial gift. Acta Zool 92: 398–403.

[35] Penny ND, Byers GW (1979) A check-list of the Mecoptera of the world. Acta Amazon 9: 365–388.

[36] Byers GW (1994) Taiwanese species of *Neopanorpa* (Insecta: Mecoptera: Parnorpidae). Ann Carnegie Mus 63: 185–192.

[37] Hua BZ, Chou I (1997) The Panorpidae (Mecoptera) of Funiu Mountain in Henan Province. Entomotaxonomia 19: 273–278.

[38] Byers GW (1965) The Mecoptera of Indo-China. Pac Insects 7: 705–748.

[39] Chau HC-S, Byers GW (1978) The Mecoptera of Indonesia: genus *Neopanorpa* . Univ Kans Sci Bull 51: 341–405.

[40] Issiki S (1933) Morphological studies on the Panorpidae of Japan and adjoining countries and comparison with American and European forms. Jpn J Zool 4: 315–416.

[41] Mickoleit G (1971) Zur phylogenetischen und funktionellen Bedeutung der sogenannten Notalorgane der Mecoptera (Insecta, Mecoptera). Z Morphol Tiere 69: 1–8.

[42] Cheng FY (1957) Revision of the Chinese Mecoptera. Bull Mus Comp Zool 116: 1–118.

[43] Arnqvist G, Thornhill R (1998) Evolution of animal genitalia: patterns of phenotypic and genotypic variation and condition dependence of genital and non-genital morphology in water strider (Heteroptera: Gerridae: Insecta). Genet Res 71: 193–212.

[44] Hosken DJ, Stockley P (2004) Sexual selection and genital evolution. Trends Ecol Evol 19: 87–93. 16701234 10.1016/j.tree.2003.11.012

[45] Miyaké T (1913) Studies on the Mecoptera of Japan. J Coll Agric Impl Univ Tokyo 4: 265–400.

[46] Crampton GC (1922) The genitalia of male Diptera and Mecoptera compared with those of related insects, from the standpoint of phylogeny. Trans Am Entomol Soc 48: 207–225.

[47] Crampton GC (1931) The genitalia and terminal structures of the male of the archaic mecopteron, *Notiothauma reedi*, compared with related Holometabola from the standpoint of phylogeny. Psyche 38: 1–21.

[48] Grell KG (1942) Der Genitalapparat von *Panorpa communis* L. Ein weiterer Beitrag zur Anatomie und Histologie der Mecopteren. Zool Jahrb Anat Ontog Tiere 67: 513–588.

[49] Byers GW (1954) Notes on North American Mecoptera. Ann Entomol Soc Am 47: 484–510.

[50] Mickoleit G (1975) Die Genital- und Postgenitalsegmente der Mecoptera-Weibchen (Insecta, Holometabola) I. Das Exoskelet. Z Morphol Tiere 80: 97–135.

[51] Mickoleit G (1976) Die Genital- und Postgenitalsegmente der Mecoptera-Weibchen (Insecta, Holometabola) II. Das Dach der Genitalkammer. Zoomorphologie 85: 133–156.

[52] Willmann R (1981) Das Exoskelett der männlichen Genitalien der Mecoptera (Insecta) II. Die phylogenetischen Beziehungen der Schnabelfliegen-Familien. Z Zool Syst Evol-forsch 19: 153–174.

[53] Willmann R (1981) Das Exoskelett der männlichen Genitalien der Mecoptera (Insecta) I. Morphologie. Z Zool Syst Evol-forsch 19: 96–150.

[54] Ma N, Zhong W, Hua BZ (2010) Genitalic morphology and copulatory mechanism of the scorpionfly *Panorpa jilinensis* (Mecoptera: Panorpidae). Micron 41: 931–938. 20702097 10.1016/j.micron.2010.07.008

[55] Engqvist L, Sauer KP (2003) Influence of nutrition on courtship and mating in the scorpionfly *Panorpa cognata* (Mecoptera, insecta). Ethology 109: 911–928.

[56] Thornhill R (1973) The morphology and histology of new sex pheromone glands in male scorpionflies, *Panorpa* and *Brachypanorpa* (Mecoptera: Panorpidae and Panorpodidae). Grt Lakes Entomol 6: 47–55.

[57] Thornhill R (1979) Male pair-formation pheromones in *Panorpa* scorpionflies (Mecoptera: Panorpidae). Environ Entomol 8: 886–888.

[58] Kock D, Ruther J, Sauer KP (2007) A male sex pheromone in a scorpionfly. J Chem Ecol 33: 1249–1256. 17497201 10.1007/s10886-007-9304-3

[59] Kullmann H, Sauer KP (2009) Mating tactic dependent sperm transfer rates in *Panorpa similis* (Mecoptera; Panorpidae): a case of female control? Ecol Entomol 34: 153–157.

[60] Kullmann H, Sauer KP (2005) Life histories and mating system aspects of two Caucasian scorpionfly species: *Panorpa similis* Esben-Petersen and *Panorpa connexa* MacLachlan. Zool Anz 244: 1–9.

[61] Thornhill R (1992) Fluctuating asymmetry and the mating system of the Japanese scorpionfly, *Panorpa japonica* . Anim Behav 44: 867–879.

[62] Kock D, Engels S, Fritsche C, Sauer KP (2009) Sexual coercion in *Panorpa* scorpionflies? –The function of the notal organ reconsidered. Behav Ecol 20: 639–643.

[63] Hua BZ (1997) A new species of the genus *Panorpa* (Mecoptera: Panorpidae) from northeast China. Entomotaxonomia 19: 213–215.

[64] Byers GW (1963) The life history of *Panorpa nuptialis* (Mecoptera: Panorpidae). Ann Entomol Soc Am 56: 142–149.

[65] Ma N, Zhong W, Gao QH, Hua BZ (2012) Female genital plate diversity and phylogenetic analyses of East Asian Panorpidae (Mecoptera). Syst Biodiver 10: 159–178.

[66] Sirot LK (2003) The evolution of insect mating structures through sexual selection. Fla Entomol 86: 124–133.

[67] Snodgrass RE (1957) Revised interpretation of external reproductive organs of male insects. Smithson Misc Collect 135: 1–60.

[68] Eberhard WG, Cordero C (2003) Sexual conflict and female choice. Trends Ecol Evol 18: 438–439. 10.1016/s0169-5347(00)89205-8 21237123

[69] Stockley P (1997) Sexual conflict resulting from adaptations to sperm competition. Trends Ecol Evol 12: 154–159. 21238013 10.1016/s0169-5347(97)01000-8

[70] Parker GA (2006) Sexual conflict over mating and fertilization: an overview. Philos Trans R Soc Lond B 361: 235–259. 16612884 10.1098/rstb.2005.1785 PMC1569603

[71] Eberhard WG, Ramirez N (2004) Functional morphology of the male genitalia of four species of *Drosophila*: failure to confirm both lock and key and male-female conflict predictions. Ann Entomol Soc Am 97: 1007–1017.

